# Advanced applications of sustainable and biological nano-polymers in agricultural production

**DOI:** 10.3389/fpls.2022.1081165

**Published:** 2023-01-06

**Authors:** Kari Vinzant, Mohammad Rashid, Mariya V. Khodakovskaya

**Affiliations:** Department of Biology, University of Arkansas at Little Rock, Little Rock, AR, United States

**Keywords:** nanotechnology, agriculture, biodegradable polymers, sustainable nanotechnology, biopolymers, Polymeric Nanoparticles

## Abstract

Though still in its infancy, the use of nanotechnology has shown promise for improving and enhancing agriculture: nanoparticles (NP) offer the potential solution to depleted and dry soils, a method for the controlled release of agrochemicals, and offer an easier means of gene editing in plants. Due to the continued growth of the global population, it is undeniable that our agricultural systems and practices will need to become more efficient in the very near future. However, this new technology comes with significant worry regarding environmental contamination. NP applied to soils could wash into aquifers and contaminate drinking water, or NP applied to food crops may carry into the end product and contaminate our food supply. These are valid concerns that are not likely to be fully answered in the immediate future due to the complexity of soil-NP interactions and other confounding variables. Therefore, it is obviously preferred that NP used outdoors at this early stage be biodegradable, non-toxic, cost-effective, and sustainably manufactured. Fortunately, there are many different biologically derived, cost-efficient, and biocompatible polymers that are suitable for agricultural applications. In this mini-review, we discuss some promising organic nanomaterials and their potential use for the optimization and enhancement of agricultural practices.

## 1 Introduction

The recent 2022 United Nations report on the world population estimates that there will be an addition of 0.5 billion people by 2030 (United Nations Department of Economic and Social Affairs, Population Division, 2022). The Food and Agriculture Organization noted that most regions experienced a decline in cropland area per capita, relating to the sharp population increase over the past two decades (FAO, 2022). These factors combined suggest that existing methods of farming need to become more efficient, as regional populations climb and threaten to surpass what arable land is available (FAO, 2022). This demand for increased productivity without expanding agricultural lands could be achieved with the use of nanotechnology. Nanotechnology can address several agricultural issues such as the controlled release of active compounds (Singh et al., 2021), selective targeting of pests (Hao et al., 2020; Monteiro et al., 2021), efficient delivery of fertilizers (Guilherme et al., 2010; Ekanayake and Godakumbura, 2021), photoprotection of light-sensitive herbicides (Nguyen et al., 2012), and a way to remediate depleted or dry soils (Kathi et al., 2021; Barajas-Ledesma et al., 2022), but have yet to see widespread practical use. The hesitation surrounding nano-enabled agriculture stems from the many “unknowns” concerning nanomaterials in the environment: what is their fate in soil (Singh and Gurjar, 2022)? will nanomaterials bioaccumulate within plants and potentially harm consumers (Murali et al., 2022)? will soil microbe communities be harmed or changed (Ameen et al., 2021)? can these materials wash into and contaminate local watersheds (Batley et al., 2013)? No complete answers for these concerns currently exist, but there is still the pressing need for both increased productivity and responsible field trials to further our understanding of nanoparticle interactions in the environment. Going forward, it is crucial that research treads carefully when applying nanomaterials in an open environment. Engineered nanomaterials are popular subjects of research, but these materials are not easily biodegradable and may persist in soils (Du et al., 2011; Liang et al., 2017; Yuan et al., 2017; Courtois et al., 2021). An alternative to such materials may be found in the form of biodegradable polymers. Though synthetic polymers have shown potential as agricultural tools, the current controversy concerning environmental microplastic contamination could sour the public opinion of these nanotechnologies, and potentially result in a similar situation to GMO crops (Sylvester et al., 2009; Grillo et al., 2012; Accinelli et al., 2019; Chen and Wang, 2019; Wang et al., 2019; Deng et al., 2020; Li et al., 2020; Brewer et al., 2021; Garcia-Vazquez and Garcia-Ael, 2021). There have been recent efforts investigating biological polymers such as polysaccharides, proteins, and even nucleic acids, for their use in the creation of nanoparticles (NP) for agriculture. These materials are great options for field applications due to their biocompatibility, biodegradability, relative abundance, and affordability. In recent research, biopolymeric NPs of different chemistries have been used to improve the uptake of plant fertilizers such as urea (Mohammadbagheri et al., 2021), encapsulation of pesticides and herbicides (Li N. et al., 2021), controlled release of fertilizers (Vejan et al., 2021), soil conditioning for enhanced water retention (Chang et al., 2021), and for post-harvest food packaging purposes such as preserving agents and films (Neme et al., 2021). Below, we characterize and highlight some promising biological polymers and recent evidence supporting the possibility of utilizing them as NP in agriculture.

## 2 Classes of biological nano-polymers

**Figure 1:**
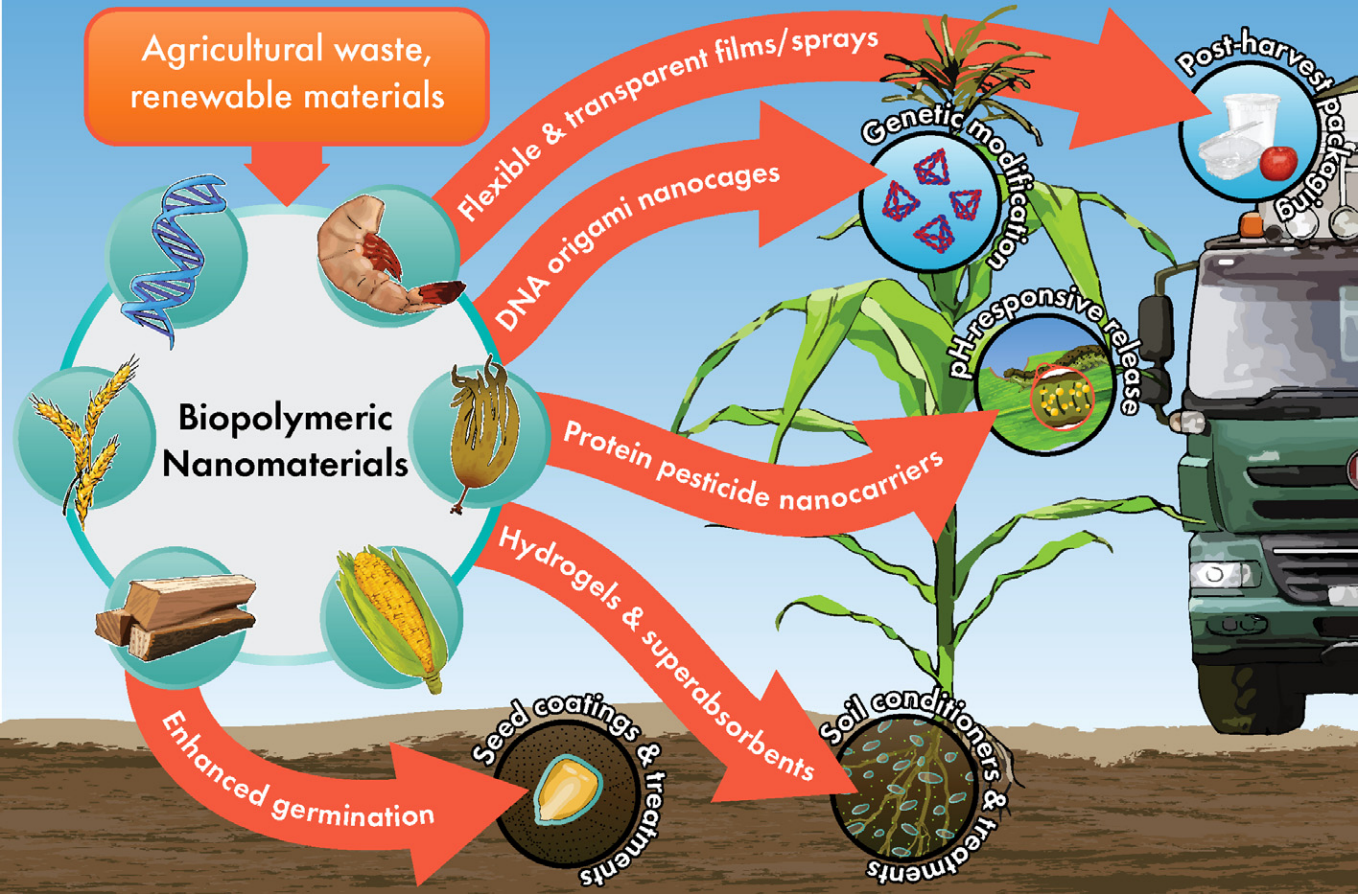
Potential agricultural applications of biopolymeric nanoparticles.

The types of biopolymers currently being researched for agricultural applications typically are either polysaccharides, proteins, or as of very recently, nucleic acids. These materials may be obtained from a variety of sources, but in an effort to keep things sustainable and cost-effective, it is best to derive NP from waste products and renewable materials. There are many different agricultural needs to which polymeric NP may be applied, as illustrated in **Figure 1**. Additional consideration should be given to dietary preference and restriction, which gives some selection against animal-derived proteins for applications regarding food crops. Therefore, we have limited our focus to several polymeric nanomaterials that shine in terms of affordability, biocompatibility, and efficiency as NP. **Table 1** details the physiochemical properties, source materials, and sizes of the NP we briefly introduce below.

**Table 1:** Physiochemical descriptions of discussed biologically-derived polymeric nanoparticles and their applications in agriculture.

Name	Proposed Agricultural Application	Molecular Class	Material Source	Zeta Potential (mV)	Average Particle Size (nm)	Solubility	Ref
Cellulose (Nanofibers)	Superabsorbent hydrogels for dry soil remediation	Polysaccharide	Agricultural waste residues such as wood pulp, wheat bran, and corn stalks	–4.6 ± 1.2	12.52 ± 8.43 wide, several micrometers long	Insoluble in water without modification	(Li et al., 2015)
Lignin (Nanocapsules)	Soil treatments as an antifungal carrier	Polysaccharide	Wood pulp	−35.4	162.4 diameter	Insoluble in water without modification	(Luo et al., 2020)
Arabinoxylan	Post-harvest packaging and films	Polysaccharide	Wheat bran	−73.6 ± 4.1 23.8 ± 0.5	93.25 ± 19.24 125.08 ± 25.83	Soluble in DMSO and water	(Sarker et al., 2020)
Pectin	Seed coatings and seed treatments	Heteropolysaccharide	Orange peels, apple pomace	− 29.6 ± 0.5	419.3	Soluble in acidic solutions, ethanol, ethyl-ether, water	(Li R. et al., 2021)
Chitosan-Alginate (Composite)	Seed treatments and plant hormone nanocarriers	Polysaccharide	Brown seaweeds, crustacean shells	32.2 ± 0.8	84 ± 8	Chitosan: soluble in water Alginate: Insoluble in water	(Pereira et al., 2017)
Zein	Insecticide nanocarriers	Protein	Corn	−34.30 ± 2.11	174.50 ± 9.79	Soluble in aqueous alcohol, high concentrations of urea, alkaline pH (>11), or anionic surfactants.	(Anderson and Lamsal, 2011)
DNA Nanostructures	Plant genetic modification	Nucleic Acid	Nucleotides, PCR	Not Applicable	Tetrahedron: 2 HT: 2x5x16 Nanostring: 2x5x320	Water soluble	(Zhang et al., 2019)

### 2.1 Polysaccharides

Polysaccharides are versatile polymers for use in NP as they may carry several different functional groups, enabling a variety of chemical methods for surface modification (da Silva Perez et al., 2003; Tizzotti et al., 2010; Meng and Edgar, 2016). Polysaccharides may be used either alone or in conjunction with other polymers to create NP. Plant waste materials such as wheat bran, wood pulp, corn stalks, and orange peels may be processed into several different polysaccharides including cellulose, lignins, xylans, and pectins (George and Sabapathi, 2015; Huang et al., 2017; Xiao et al., 2019; Chandel et al., 2022). Popular non-plant-derived nanomaterials include chitosan and alginate. Alginate is derived from brown seaweeds, while chitosan is derived from crustacean shells and fungi (Paques et al., 2014; Ahmed and Aljaeid, 2016). Except for chitosan, polysaccharide NPs typically possess negative charges without surface modification.

### 2.2 Proteins

There are several unique advantages to using proteins in nanoparticle design such as matrix self-assembly, but one of the most significant is the ability to design a selective release system that is responsive to a unique factor, such as pH, or even enzymatic activity (Jain et al., 2018). Typically designed around a protein matrix core and polysaccharide or lipid shell, these systems utilize the protein matrix to trap a compound of interest, and rely on the disruption of that matrix to trigger the quick release of contents, whether by a change in pH, disruption of disulfide bridges, or enzymatic cleavage (Fathi et al., 2018; Jain et al., 2018). Though there are several plant-based proteins currently being investigated, for agriculture-centric research the most popular by far is zein. Zein is the primary protein present in corn, with it accounting for 50% of all protein content in corn. Zein is a prolamine possessing four isoforms, with α-zein being the most abundant (Li and Yu, 2020). Some other plant-based proteins of interest in research include gliadins from wheat, various proteins from soy, peanut proteins, and pea proteins (Teng et al., 2012; Vo Hong et al., 2016; Shi et al., 2017; Doan and Ghosh, 2019).

### 2.3 Nucleic acids

DNA origami has gained some recent attention as a potential method of delivering genetic material into eukaryotic cells. DNA origami takes advantage of the classic Watson-Crick base pairing to design complex 3D shapes with DNA (Zhang et al., 2020). These DNA nanoforms are designed using computer software such as caDNAno or Tiamat, and have been used to engineer molecular machines, which can utilize input logic gates based on aptamer conformation, or be used as vessels for drug and biomolecule delivery (Dey et al., 2021). DNA nanoforms are attractive options as delivery vehicles for DNA, RNA, and proteins as the nanostructure itself may be designed to include loci that associate with the desired cargo. Appropriate DNA origami designs may present a way of introducing caged materials into plants.

## 3 Applications of nano-biopolymers

### 3.1 Insecticides

Insect damage on crops is estimated to cause up to 40% of global crop loss annually (IPPC Secretariat., 2021). Additionally, many crop diseases, such as citrus greening disease and potato leaf roll virus can be spread by insect carriers (Heck, 2018). Therefore, it is a common agricultural practice to employ the use of insecticides. Compared to acidic mammalian guts, Lepidopteran species are known to have alkaline guts, which is a trait that can be selectively targeted using protein pesticide delivery systems (Dow, 1992). Zein is an appealing choice of pesticide carrier due to its affordability, willingness to self-assemble, and surface charge flexibility (Jain et al., 2018). Zein is appropriate for use with plants and has been shown to be even safer for plants than unencapsulated pesticidal agents (de Oliveira et al., 2018; Pascoli et al., 2020). Zein is an easy choice of pesticide carrier targeting Lepidopteria species, as many high-profile pests from this family naturally feed on corn (Reay-Jones, 2019). Zein NPs have been shown to release their contents when incubated with fall armyworm (*Spodoptera frugiperda)* larval midgut extracts, suggesting selective targeting of the insect gut is possible (Monteiro et al., 2021). Zein may additionally be modified to enhance uptake into plant systems. Zein NP treated with SDS to convey a negative surface charge were loaded with methoxyfenozide (MFZ), and shown to have greater uptake and translocation than free MFZ in hydroponically-grown soybean (*Glycine max)* (Hanna et al., 2022). MFZ levels in roots, shoots, and leaves were higher than in control at lower doses and continued to accumulate over 24 hours, whereas the levels in plants treated with free MFZ tapered off over time (Hanna et al., 2022). Zein NP translocation to soybean leaves was significantly more efficient than that of free MFZ, which is of particular interest as soybean looper (*Chrysodeixis includens)*, a major pest of the crop, feeds on leaf tissues. Authors suggest this method of delivering pesticides through the roots may have advantages over traditional sprays: there may be less of a need for repeat applications, and less risk of environmental toxicity (Hanna et al., 2022). These are promising results demonstrating zein NP insecticidal potential as several caterpillar species are significant threats to corn, cotton, and soybean crops.

### 3.2 Soil conditioners and treatments

Lignocellulosic NPs possess numerous hydroxyl groups, which give them both the ability to create strong hydrogen bonds with water and also be excellent candidates for surface modification (Lam et al., 2012). These attributes allow them to form hydrogels, which are useful for soil conditioning. Hydrogels and superabsorbent materials can slowly release water into the surrounding environment and can be useful for prolonging water retention in sandy soils (Narjary et al., 2012).

Cellulose nanofibers (CNF) derived from (2,2,6,6-Tetramethylpiperidin-1-yl)oxyl (TEMPO) mediated oxidation have been shown to increase water retention for a longer time compared to control soils. Soils remedied with a 1.0 wt% superabsorbent exhibited slower water evaporation than control, delaying wilting up to 20 days, suggesting that CNF superabsorbents may have use in soil conditioning and prolonging water retention in dry soils (Barajas-Ledesma et al., 2022). Cellulosic NP may also be used in conjunction with inorganic NP such as iron to improve the loading efficiency of hydrogels and provide prolonged, consistent release of fertilizers. One such hydrogel based on TEMPO CNF and a metal-organic framework (MOF) composed of MIL-100(Fe) was demonstrated to be an appealing opportunity for the slow release of urea, a common fertilizer as a source of nitrogen. These hydrogels were shown to be both pH and temperature responsive, with urea releasing slower at pH 11, and releasing faster at higher temperatures (45°C). Plants treated with CNF-MOF formulations produced more biomass over 60 days compared to those treated with free urea (Lin et al., 2021).

Lignocellulosic NPs can also be used to enhance soil treatments by providing a negative charge to the NP surface. Loaded lignin-modified nanocapsules were recently demonstrated to have potential as a soil treatment against Fusarium crown and root rot, diseases caused by pathogenic fungi in the soil that can infect and hinder the development of young seedlings. Pyraclostrobin is a fungicide that has high efficacy against *Fusarium oxysporum* in the lab but has lower success in the field due to the adsorbents in the soil (Reddy et al., 2013). Using sodium lignosulfonate as a nanocarrier, a negatively-charged shell can be formed around the pyraclostrobin and increase soil mobility (Luo et al., 2020). Formulations using lignosulfonate had better soil mobility than nanoemulsions in water, and nanocapsule-treated plants had the least amount of crown and root rot compared to control and other treatments (Luo et al., 2020).

### 3.3 Seed coatings and treatments

Seed coating is the practice of covering the outer surface of a seed with a material to protect and nurture seedlings shortly after they are planted. Pectin has been shown to have some antifungal properties, which makes it a material of interest for seed coatings (Ciriminna et al., 2020). Nanopectin treatments are not harmful to seed germination and may even enhance seedling biomass (Li R. et al., 2021). A pectin-neem oil nanocomposite seed coating was recently shown to promote germination in soybean seeds and exhibited antifungal properties against *Aspergillus flavus* and *Penicillium citrinum* (de Castro e Silva et al., 2019). In addition to finding evidence of antifungal activity in their pectin-neem oil nanocomposites, the work also notes that seedlings germinated from seeds that were treated with a 70:30 pectin:neem oil formulation had earlier germination and longer shoots compared to other formulations (de Castro e Silva et al., 2019).

Chitosan (CS) NP loaded with garlic essential oil was shown to be an effective antifungal treatment for cereal grains including wheat (Mondéjar-López et al., 2022). Wheat seedlings that germinated from seeds treated with a 7.6 mg/mL NP concentration had significantly greater total weights and root biomass compared to those treated with the recommended dose of tebuconazole, a commercial antifungal (Mondéjar-López et al., 2022). Germination assays determined that seeds coated with 2.5 mg/mL NP formulations experienced no phytotoxicity from the treatment. Antifungal assays against *Fusarium oxysporum*, *Aspergillus niger*, and *Aspergillus versicolor* were also successful at a similar dose of 7.5 mg/mL. With all evidence combined, this NP formulation might present a greener alternative to the commercial option of tebuconazole (Mondéjar-López et al., 2022).

CS-based NPs can also be used as nanocarriers for plant growth regulators as a form of seed priming (Pereira et al., 2017). Gibberellic acid (GA3) is a plant hormone often used to break seed dormancy and promote growth (Hedden and Sponsel, 2015). Pea (*Phaseolus vulgaris*) seedlings grown from seeds treated with a CS/tripolyphosphate NP carrying GA3 showed greater root biomass at lower concentrations compared to free GA3, and developed greater leaf areas compared to controls (Pereira et al., 2017). In the same investigation, authors also create NP using alginate (ALG) and CS. Though not the focus of the study, authors noted that plants treated with ALG/CS containing no GA3 grew surprisingly similar to that of free GA3. The ALG/CS had been prepared using a solution of CaCl_2_, which has been positively associated with plant growth when applied exogenously, partially because of its influence on GA3 and indole-acetic acid levels (Wang et al., 2016). Two years later, the authors would conduct a second study using tomato (*Solanum lycopersicum*) and similar CS/Tripolyphosphate and ALG/CS nanocarriers for GA3. Dilute treatments of GA3-loaded ALG/CS NP solutions resulted in significantly higher root and shoot biomass in treated seedlings (Pereira et al., 2019). There is an apparent synergistic effect between GA3 and NP formed in CaCl_2_ solution, which may make the cost-effective ALG/CS nanocarriers an appealing choice in seed priming.

### 3.4 Post-harvest packaging materials

There is substantial interest in biodegradable and renewable food packaging materials to replace the plastics currently being used. Xylans show excellent promise for use as transparent films, but require modification or a copolymer to be most effective at forming a flexible film (Zhong et al., 2013; Zhang et al., 2018). Arabinoxylan and nonfibrillated cellulose films have been shown to be a potential substitute for petroleum-based films for food packaging (Stevanic et al., 2012). Xylan-ALG films reinforced with CNF showed better tensile strength and lower water vapor permeability than non-reinforced films (Naidu and John, 2021). Transport of fruits and vegetables is crucial to make the food available almost everywhere therefore post-harvest storage is a concern. Composites of arabinoxylan and β-D-glucan stearic acid ester have been proven to extend the shelf life of peach fruits while maintaining their nutritional quality (Ali et al., 2021).

### 3.5 Plant genetic modification

As the transformation of plant cells is currently reliant on *Agrobacterium tumefaciens* or particle gun bombardment, there is a need for a more elegant method for the reliable transformation of plant cells. The rigid cell walls of plant cells are particularly hard to bypass and will prevent the uptake of particles larger than 5-20 nm, with the internal cell membrane being permeable to particles smaller than 500 nm (Cunningham et al., 2018). NPs that meet these size requirements may find entry into plant vascular systems through the roots or stomata (Hu et al., 2020; Li et al., 2020). If organic NPs are absorbed by the roots, they may be taken from the roots through the xylem, and circulated through the plant, potentially entering reproductive tissues (Prasad et al., 2018; Li et al., 2020; Rezaei Cherati et al., 2022). This systematic penetration is highly desirable for plant genetic modification.

There is currently more research available on DNA origami and its use in animal cells, but some early investigations show promise in using DNA origami to deliver siRNA into plant cells. In their 2019 publication, Zhang et al. explored the efficacy of differently-shaped DNA nanostructures to deliver siRNA targeting GFP in tobacco leaves (Zhang et al., 2019). 21-bp siRNA segments were loaded into DNA tetrahedrons, hairpin-tiles (HT) loaded from the center and from the side, and nanostrings before being injected into tobacco leaves with a needleless syringe. All siRNA-laden DNA nanostructures performed better than naked siRNA, with side-loaded HT offering the highest silencing efficiency. The higher silencing efficiency of the side-loaded HT is thought to be a result of the greater degree of internalization, which may be due to the higher aspect ratio of the HT design compared to the tetrahedron (Zhang et al., 2019). However, the lowest amount of silencing was observed with the nanostring structures which have the highest aspect ratio out of the forms tested. Authors speculate that size alone may not be the only factor determining internalization, and rigidity of the nanostructure may also play into the rate of internalization. This is experimentally confirmed by the tethering of nanostrings to single-walled carbon nanotubes (SWCNT), which are very rigid. The authors observed higher internalization for the nanostring-SWCNT conjugate compared to naked nanostrings. Authors also identify the cost of a single infiltration using this technique as relatively cheap and potentially scalable, with simple geometries such as tetrahedrons, HT monomers, and nanostrings costing $0.53-$0.65 USD, suggesting that DNA origami techniques may be a more cost-effective alternative to other transformation options such aswith gene gun based methods (Zhang et al., 2019).

## 4 Discussion

In this mini-review, we have highlighted a few natural biopolymers suitable for use in agriculture. Ultimately, all discussed materials are appropriate for use in food and agriculture as their bulk forms are all well-characterized and considered benign (21 CFR 184.1588 – Pectins; Loh et al., 2010; Roman, 2015; Ristroph et al., 2017; Prasad et al., 2018; Ciriminna et al., 2020.; 21 CFR 184.1724 – Sodium alginate.; 21 CFR 184.1984 – Zein.). However, natural polymeric systems are not without flaw. One point of concern is regarding the use of protein-based systems. The potential for stimulating an immunogenic response for protein nanocarriers has not been well-investigated, and many types of proteins being researched are associated with food allergies. For example, though wheat proteins such as glutenins and gliadins are currently being examined for controlled release of different drugs, it is worth noting that gliadins have been implicated as triggering agents in Celiac Sprue patients (Shan et al., 2005; Sharif et al., 2018; Serena et al., 2020). Additionally, soy proteins glycinin and β-conglycinin can trigger soybean allergies (Zhao et al., 2015; Zhao et al., 2021). Zein is a comparatively low-risk protein in terms of immunogenicity. Though some people do experience corn sensitivity, evidence points to a lipid transfer protein, not zeins, as the allergenic trigger (Pastorello et al., 2000). When utilizing protein NP, it is crucial to ensure the purity of the proteins not only for the stability and function of the NP themselves but also for maintaining the safety of those who may be allergic to one or more components found within the source material. Since functional nanomaterials typically lie within the ranges of 10-100 nm in size, it is important to be aware that these sizes also are effective at eliciting immune responses for recognized antigens (Bachmann and Jennings, 2010). Overall, biopolymers are still the safer option for initial field research compared to other classes of nanomaterials, and a great starting point for pushing nano-enabled agriculture closer to actualization.

## Author contributions

All authors listed have made a substantial, direct, and intellectual contribution to the work and approved it for publication.
